# Premature Coronary Heart Disease and Traditional Risk Factors-Can We Do Better?

**Published:** 2013-06-01

**Authors:** Roxana Sadeghi, Nadia Adnani, Azam Erfanifar, Latif Gachkar, Zohre Maghsoomi

**Affiliations:** 1 Cardiovascular Research Center, Shahid Beheshti University of Medical Sciences, Tehran, IR Iran; 2 Faculty of Medicine, Loghman Hakim Hospital, Shahid Beheshti University of Medical Sciences, Tehran, IR Iran; 3 Faculty of Medicine, Shahid Beheshti University of Medical Sciences, Tehran, IR Iran

**Keywords:** Atherosclerosis, Risk Factors, Coronary Heart Disease, Coronary Artery Disease

## Abstract

**Background:**

Traditional cardiovascular risk factors are strong predictors of an increased likelihood for premature CHD. Considering the benefits of risk factors᾿ management, it is imperative to find and treat them before looking for more unknown and weak risk factors.

**Objectives:**

Limited information is available about the demographic and historical characteristics of the patients with premature Coronary Heart Disease (CHD) in IR Iran. The main objective of this study was to determine the prevalence of the traditional risk factors in these patients. Also, the researchers hypothesized that there are insufficient risk assessment and preventive intervention methods for the asymptomatic adult population.

**Methods:**

This study was conducted on 125 patients with premature CHD (age<50 years) who were admitted in two academic hospitals with acute coronary syndromes. The patients were accepted since they had a definite CHD on the basis of acute myocardial infarction (elevated cardiac enzymes) or documented CAD in coronary angiography.

**Results:**

The mean age of the study population was 42.50±5.65 (26 to 49 years). Among the patients,92 (73.6%) were male, 113 (90.4%) were married, 58 (46.4%) were smokers,19 (15.2%) were opium users, 97 (77.6%) had dyslipidemia, 44 (35.2%) had hypertension, and 33 (26.4%) had diabetes mellitus. In addition, family history was presented in 54 patients (43.2%).

Among the study population, 120 patients (96%) had at least one of the traditional risk factors, including dyslipidemia, hypertension, diabetes mellitus, cigarette smoking, and family history of CHD. However, none of the dyslipidemic patients had controlled total cholesterol, LDL, HDL, and triglyceride. Also, none of the diabetic patients had hemoglobin A1C<7%. Among the 44 hypertensive patients, blood pressure of 15 ones (34%) was within the normal range. Besides, only 3 patients (2.4%) had regular physical activity (at least 30 minutes, three times a week).

**Conclusions:**

Premature Coronary Heart Disease is a public health problem. However, there is lack of effective and intensive treatments of well-defined traditional risk factors and prevention methods for the majority of the patients experiencing premature CHD. In sum, there is still plenty of room for improvement of risk management in IR Iran.

## 1. Background

The incidence of premature Coronary Heart Disease (CHD) is quite high in IR Iran as well as many other countries (1,2). It is necessary to know the true magnitude of this problem for improving risk stratification as well as the prevention methods in order to provide optimal care for those with or at risk of developing CHD. Multiple clinical trials have proven that appropriate detection and treatment of the risk factors can slow the progression of atherosclerosis and reduce the occurrence of cardiovascular events.

In multiple studies, traditional risk factors, such as family history of premature CHD (3-5), dyslipidemia (6), hypertension, diabetes mellitus, and cigarette smoking, have been shown to be significantly associated with early CHD.

Despite this fact, the application of primary prevention is not optimal for well-known risk factors and further risk stratification and aggressive treatment is needed. Non-traditional risk factors and inflammatory biomarkers would also be the second treatment goal; however, they are only applicable after optimal treatment of the traditional risk factors.

## 2. Objectives

The aim of this study is to show the prevalence and management of traditional risk factors in a population with premature CHD.

## 3. Patients and Methods

Among all the patients with acute coronary syndromes who had been admitted in the cardiovascular centers in two academic hospitals between January 2011 and March 2012,125 ones with definite premature CHD were selected. Coronary Artery Disease (CAD) before the age of 50 was determined as premature. The patients were identified as having definite CHD on the basis of acute myocardial infarction (elevated cardiac enzymes) or documented CAD in coronary angiography. Information about age, gender, family history of CAD (male first degree relatives<55 years old and female first degree relatives<65 years old), dyslipidemia (high LDL-cholestrol based on ATP III or HDL-cholestrol<40 mg/dL or triglycerides>150 mg/dL) (7), diabetes mellitus (fasting blood glucose≥126 mg/dL, 2 hours postprandial glucose≥200 mg/dL, or use of hypoglycemic agents or insulin), hypertension (positive past history of hypertension or use of antihypertensive drugs), smoking, and opium consumption were collected. The clinical presentations, electrocardiographic and echocardiographic results, and coronary angiographic findings were gathered and recorded, as well. A diameter stenosis>50% in each epicardial coronary artery was defined as significant CAD and a narrowing<50% was considered as mild CAD. It should be noted that written informed consents were obtained from all the study patients.

### 3.1. Statistical Analysis

Continuous variables were expressed as mean±standard deviation and dichotomous variables as frequencies. All the statistical analyses were performed using the SPSS statistical software (version 16).

## 4. Results

The mean age of the study population was 42.50±5.65 years (26 to 49 years) and 92 patients (73.6 %) were male. In addition, 58 patients (46.4%) were smokers and 19 ones (15.2%) were opium users. However, none of the study subjects used amphetamine or alcohol. Moreover, 97 (77.6%), 44 (35.2%), 33 (26.4%), and 1 (0.8%) patients had dyslipidemia, hypertension, diabetes mellitus, and renal insufficiency, respectively, while none had chronic lung disease. Besides, none of the patients presented with peripheral vascular disease, cerebrovascular disease, and prior congestive heart failure. Yet, prior CAD, prior percutaneous coronary intervention, and prior coronary artery bypass graft were detected in 4 (3.2%), 3 (2.4%), and 1 (0.8%) patients, respectively. The mean of total cholesterol, LDL, HDL, and triglyceride were 193.0±46.0 (98 to 403 mg/ dL), 113.4±41.1 (22 to 279 mg/ dL), 41.8±13.0 (20 to 100 mg/ dL), and 182.1±96.6 (35.3 to 523 mg/ dL), respectively. Likewise, the mean of non-HDL cholesterol was 151.3±46.9.

Among the study population, 116 patients (92.8%) had at least one of the traditional risk factors, including dyslipidemia, hypertension, diabetes mellitus, and cigarette smoking. Besides, 120 ones (96%) had dyslipidemia, hypertension, diabetes mellitus, cigarette smoking, or family history of CHD. Nonetheless, none of the dyslipidemic patients had controlled total cholesterol, LDL, HDL, and triglyceride. Also, none of the diabetic patients had hemoglobin A1C<7%. Among the 44 hypertensive patients, only 15 ones (34%) reached the normal blood pressure. Furthermore, regular physical activity (at least 30 minutes, three times a week) was restricted to 3 study patients (2.4%). Demographic and historical characteristics of the study patients are summarized in Table 1. 

**Table 1.** Demographic and Historical Characteristics of the Patients with Premature Coronary Heart Disease

Characteristics	Number (%) or mean± SD[Table-fn fn4951]
Age, years	42.50±5.65
Male gender	92(73.6)
Marriage	113(90.4)
Family history of CAD	54(43.2)
Smoker	58(46.4)
Opium user	19(15.2)
Alcohol	0(0)
Hypertension	44(35.2)
Dyslipidemia	97(77.6)
Elevated Cholestrol (>200mg/dL)	47(37.6)
Total Cholestrol (mg/dL)	193.0±46.0
Elevated LDL	38(30.4)
LDL (mg/dL)	113.4±41.1
Non HDL cholesterol (mg/dL)	151.3±46.9
Low HDL (<40mg/dL)	63(50.4)
HDL (mg/dL)	41.8±13.0
Elevated Triglyceride (>150mg/dL)	71(56.8)
Triglyceride (mg/dL)	182.1±96.6
Diabetes mellitus	33(26.4)
FBS (mg/dL)	110.1±39.3
Renal insufficiency	1(0.8)
Serum Cr (mg/L)	1.10±0.52
WBC (cells/µL)	8242.3±2677.2
Hemoglobin (g/dL)	14.6±9.9
Platelet (106/L)	246760±225975
Chronic lung disease	0(0)
Peripheral vascular disease	0(0)
Prior cerebrovascular disease	0(0)
Prior coronary artery disease	4(3.2)
Prior congestive heart failure	0(0)
Prior CoronaryArtery Bypass Graft	1(0.8)
Prior Percutaneous Coronary Intervention	3(2.4)
Regular physical activity (at least 30 min, 3 times per week)	3(2.4)

Values are presented as n (%) unless otherwise expressed.

The clinical presentation was unstable angina, non ST elevation Myocardial Infarction (MI), or ST elevation MI in 60.8%, 7.2%, and 32% of the patients, respectively. The first electrocardiogram showed normal findings in 35.2% of the cases and left ventricular ejection fraction was more than 50% in 72% of the patients. Moreover, minimal coronary artery disease, one-vessel disease, two-vessel disease, three-vessel disease, and left main involvement in coronary angiography were observed in 18.4%, 44%, 16%, 16%, and 0% of the subjects, respectively. Also, 5.6% of the patients had no coronary artery stenosis.Clinical and Para clinical characteristics of the enrolled patients with premature CHD are shown in Table 2. 

**Table 2.** Clinical and Para Clinical Characteristics of the Patients with Premature Coronary Heart Disease

Characteristics	Number (%)
Electrocardiogram	
Available	125(100)
Normal	44(35.2)
Clinical Presentation	
Unstable Angina	76(60.8)
Non ST elevation Myocardial Infarction	9(7.2)
ST elevation Myocardial Infarction	40(32)
LV Ejection Fraction	
Available	124(99.2)
LV Ejection Fraction≥50%	90(72)
Coronary Artery Angiogram	
Available	125(100)
Normal	7(5.6)
Minimal Coronary Artery Disease	23(18.4)
One Vessel Disease	55(44)
Two Vessel Disease	20(16)
Three Vessel Disease	20(16)
Proximal LAD[Table-fn fn4952]	23(18.4)
Left Main Coronary Disease	0(0)

Abbreviations: LAD, Left Anterior Descending; LCX, Left Circumflex; RCA, Right Coronary Artery

## 5. Discussion

The mean age and gender distribution of the patients with premature CHD in this study was similar to those of the previous reports (1). In general, family history of premature CHD is a known risk factor for cardiovascular events. Evidence supports a higher incidence of subclinical atherosclerosis in the individuals with positive familial history of premature CHD (8). Even a positive family history which is not premature should be considered important (9). Of course, the causes of this familial clustering have not been established, yet. The prevalence of a positive family history in the patients with early CAD was up to 75% in some studies; however, it was 43.2% in this study (10). Thus, familial history provides an opportunity for these asymptomatic individuals who may benefit from vascular disease screening (11).

Dyslipidemia management as emerged as a key therapeutic strategy to reduce both primary and secondary cardiovascular events. In the last European guideline, the optimal LDL-C level for the asymptomatic patients was stated as less than 100 mg/dL (12).Yet, some studies have demonstrated that less than 30% of the patients have achieved the recommended level (13). In addition, the patients with low HDL-C and/or elevated triglycerides remained at an elevated residual risk even at the recommended LDL-C targets. Hypertriglyceridemia is a significant independent predictor of CHD, but its association is not as strong as that of LDL (14). Low level of HDL cholesterol is an independent and important risk factor for coronary artery disease . The combination of moderately elevated triglycerides and low concentration of HDL cholesterol is very common in diabetic patients (15). In comparison to LDL, non-HDL cholesterol is more strongly associated with CAD risk. It is particularly more reliable than LDL calculated through the formula in the patients with hypertriglyceridemia (16). In this study, none of the dyslipidemic patients had controlled total cholesterol, LDL, HDL, and triglyceride. Thus, dyslipidemia can be another important therapeutic target for health providers.

Hypertension is one of the leading preventable causes of premature CHD and thereby death (17). Hypertensive patients more commonly have other atherosclerotic risk factors which may interact with high blood pressure. Thus, hypertensive patients are at increased risk despite the mild or moderate elevation in blood pressure. Therapeutic lifestyle changes and pharmacologic interventions are mandatory for controlling hypertension. However, only 34% of the hypertensive patients achieved controlled blood pressure in this study.

Furthermore, diabetic patients have a two- to four- fold increased risk for development of CAD and death (18). In diabetic patients, intensive management of hyperglycemia reduces the percentage of microvascular complications and, to a lower degree, the percentage of macrovascular complications. Overall, the target HbA1C in diabetic patients is<7% after the treatment; however, none of the diabetic patients had hemoglobin A1C<7% in this study.

Smoking is the most important risk factor for vascular disease worldwide. Some studies have shown that 40% of all heart diseases are related to smoking (19). Also, evidence has demonstrated that smoking cessation is associated with significant reduction in the risk of CHD, cerebrovascular diseases, and cancers. The relative risk of heart attack in the smokers below 50 years old is five folds higher than that of the non-smokers, while this risk is only doubled in the smokers above 60 years old (20).

Passive smoking also increases the risk of CAD more than it might be expected (21). Therefore, behavioral skills and pharmacologic treatments are recommended to facilitate smoking cessation (22). In the current study, 46.4% of the patients with premature CHD were smokers and none of them had successful smoking cessation.

Among the study subjects, 120 ones (96%) had at least one of the risk factors, including dyslipidemia, hypertension, diabetes mellitus, cigarette smoking, and family history of CHD. In other studies also, 90% of the patients with CHD had one atherosclerotic risk factor (23).

Regular physical activity reduces the risk of fatal and non-fatal coronary events in not only the healthy individuals, but also the patients suffering from atherosclerotic risk factors (24). Hence, guidelines have recommended physical activity as a very effective non-pharmacological tool in primary prevention (12). In the present study, regular physical activities were restricted to 2.4% of the patients.

CHD with its long asymptomatic latent period provides an opportunity for early effective preventions (25).

Atherosclerotic cardiovascular disease, especially CHD, is the leading cause of death worldwide; however, the mortality rate can be significantly reduced (more than 50%) with good primary prevention of CHD (12).

## 6. Conclusion

Traditional risk factors, such as dyslipidemia, hypertension, diabetes, and smoking, are significantly related to premature CHD. However, the researchers assume that full treatment of known and well-defined risk factors was not accomplished in this study.

**Study limitations**

The present study had some limitations. First, the data regarding weight, height, and body mass index were incomplete. Furthermore, physical activity as a part of the occupational work of the patients was not taken into consideration. Second, self-reported family histories might be potentially impacted by recall bias. In the previous studies, the positive predictive value for self-reports of premature CHD was low, while the negative predictive value was high (26). Finally, further studies are needed to be conducted on a larger number of patients in order to arrive at more reliable findings.
